# Novel Two-Dimensional Carbon–Chromium Nitride-Based Composite as an Electrocatalyst for Oxygen Reduction Reaction

**DOI:** 10.3389/fchem.2019.00738

**Published:** 2019-11-12

**Authors:** Karim Khan, Ayesha Khan Tareen, Muhammad Aslam, Qasim Khan, Sayed Ali Khan, Qudrat Ullah Khan, Awais Siddique Saleemi, Renheng Wang, Yupeng Zhang, Zhongyi Guo, Han Zhang, Zhengbiao Ouyang

**Affiliations:** ^1^Key Laboratory of Optoelectronic Devices and Systems of Ministry of Education and Guangdong Province, THz Technical Research Center, College of Physics and Optoelectronic Engineering, Shenzhen University, Shenzhen, China; ^2^Shenzhen Engineering Laboratory of Phosphorene and Optoelectronics, and SZU-NUS Collaborative Innovation Center for Optoelectronic Science and Technology, Shenzhen University, Shenzhen, China; ^3^Advanced Electromagnetic Function Laboratory, Dongguan University of Technology, Dongguan, China; ^4^Government Degree College PaharPur, Gomel University, Dera Ismail Khan, Pakistan; ^5^Shenzhen Key Laboratory of Flexible Memory Materials and Device, College of Electronic Science and Technology, Shenzhen University, Shenzhen, China; ^6^Key Laboratory of Optoelectronic Devices and Systems of Ministry of Education and Guangdong Provence, College of Optoelectronic Engineering, Shenzhen University, Shenzhen, China

**Keywords:** two-dimensional binary oxide/nitride composite, reduced graphene oxide, nitration, oxygen reduction reaction applications in fuel cells, more active and highly stable catalyst

## Abstract

For future pollution-free renewable energy production, platinum group metal (PGM)-free electrocatalysts are highly required for oxygen reduction reaction (ORR) to avoid all possible Fenton reactions and to make fuel cell more economical. Therefore, in this study, to overcome traditional electrocatalyst limitations, we applied facile method to synthesize robust mesoporous CrN-reduced graphene oxide (rGO) nanocomposite with MnO (thereafter, Cr/rGO composite with MnO) as an electrocatalyst by efficient one-step sol-gel method by ammonolysis at 900°C for 9 h. Synthesized porous structures of Cr/rGO nanocomposite with MnO have the highest estimated surface area of 379 m^2^·g^−1^, higher than that of the carbon black (216 m^2^·${\text{g}}_{\text{cat}}^{-1}$) support, and almost uniform pore size distribution of about 4 nm. The Cr/rGO nanocomposites with MnO exhibit enhanced electrocatalytic ORR properties with estimated high half-wave potential of 0.89 V vs. the reversible hydrogen electrode (RHE) and current density of 5.90 mA·cm^−2^, compared with that of benchmark 20% Pt/C electrode (0.84 V, 5.50 mA·cm^−2^), with noticeable methanol tolerance and significantly enhanced stability in alkaline media. Hence, the Cr/rGO nanocomposites with MnO showed superior performance to 20 wt.% Pt/C; their half-wave potentials were 50 mV high, and the limiting current density was 0.40 mA·cm^−2^ high. In alkaline anion exchange membrane fuel cell (AAEMFC) setup, this cell delivers a power density of 309 mW·cm^−2^ for Cr/rGO nanocomposite with MnO, demonstrating its potential use for energy conversion applications. The nanosized Cr/rGO metallic crystalline nanocomposites with MnO gave a large active surface area owing to the presence of rGO, which also has an effect on the charge distribution and electronic states. Hence, it may be the reason that Cr/rGO nanocomposites with MnO, acting as more active and more stable catalytic materials, boosted the electrocatalytic properties. The synergistic consequence in nanosized Cr/rGO composite with MnO imparts the materials' high electron mobility and thus robust ORR activity in 0.1 M of KOH solution. This potential method is highly efficient for synthesis of large-scale, non-noble-metal-based electrocatalytic (NNME) materials (i.e., Cr/rGO nanocomposite with MnO) on the gram level and is efficient in preparing novel, low-cost, and more stable non-PGM catalysts for fuel cells.

## Introduction

The increasing global energy demand along with fossil fuel depletion-based production causes environmental concern and has stimulated intensive research on clean and sustainable energy production (Li et al., 2016; Guo et al., 2017; Ren et al., 2017; Khan et al., 2018d; Walter et al., 2018). Fuel cell is possibly an alternative to the fossil fuel-based energy owing to its high power density and zero emission of greenhouse gases. The fuel-cell technology will extremely assist in transportation and diverse applications, enabling energy security and resiliency. Proton-exchange membrane fuel cells (PEMFCs) mainly use platinum group metal (PGM)-based catalysts, which are more expensive and unstable, restricting the economical and global production of PEMFCs. Thus, alkaline anion exchange membrane fuel cells (AAEMFCs) were initiated. By switching from acidic to alkaline electrolyte, it is possible to use non-noble-metal-based electrocatalyts (NNMEs) for cathode to overcome the requirements of large amount of Pt in acid medium.

Previously used commercial electrodes based on PGM catalysts suffered from a number of drawbacks including scarcity and consequent high cost, less robustness, easily poisoned, and low methanol tolerance. Another obstacle by using PGM catalysts was sluggish oxygen reduction reaction (ORR) kinetics at the cathode, which was considered as a major issue that causes a decrease in efficiency of energy-conversion devices. Cathode materials based on PGMs also provide complicated operating conditions that make them sufficiently durable to permit commercial deployment (Khan et al., 2018d). In addition, the carbon black with PGMs was used as a support material, but it corrodes too rapidly under fuel-cell operating conditions, and hence, the fuel cell efficiency decreases (Subban et al., 2010). Therefore, the synthesis of the some new electrocatalysts that do not contain PGMs, as well as iron catalysts, and free of extra carbon black addition, are highly desirable for ORR. Thus, to facilitate possible ORR with appropriate potential, the introduction of electrocatalysts is a key issue till now. Researchers in fuel-cell technology are paying attention on advancing existing synthesis technique or innovating facile synthesis scheme to develop new, highly stable/efficient electrode materials for ORR in fuel cells. In this regard, Cr- and Mn-based catalysts have drawn our attention (Walter et al., 2018).

For developing new electrocatalysts, some important necessities should be fulfilled, like high surface area, excellent conductivity, and low corrosion with good electrochemical stability in operating conditions of fuel cell. So among non-precious ORR cathode catalyst materials, nitrides along with oxides are the most promising options. This research area is being driven by a need to replace scarce noble-metal-based catalysts in energy technologies. Thus, in this work, we focused on a nitride/oxide-based catalyst to further simplify fuel-cell operation and lower the cost. In addition to this, another important factor regarding further boosting of catalyst properties is the introduction of the two-dimensional (2D) materials (Lu et al., 2013; Bai et al., 2015; Ma et al., 2015; Wan et al., 2015; Huang et al., 2016; Jiang et al., 2016; Wang et al., 2016; Zhang et al., 2016; Sun et al., 2017), particularly graphitized carbon (e.g., the rGO) with electrocatalysts (Bai et al., 2015). Recently, the 2D honeycomb graphene family with sp^2^-hybridization is a potential candidate for the ORR for its large surface area, good electrical conductivity, and excellent mechanical properties (Khan et al., 2019a). These heteroatom-doped carbon-based materials are potential “Pt” alternatives for highly efficient ORR. In fact, quantum chemical calculation revealed that the difference in electronegativity between the heteroatom dopants (N = 3.04, B = 2.04, P = 2.19, S = 2.58, Cl = 3.16, Br = 2.96, and I = 2.66) and carbon atom (2.55) in covalently doped graphitic carbon frameworks can probably polarize adjacent carbon atoms to create a net positively/negatively charged centers, which can potentially facilitate the ORR process (Liu et al., 2015). It is significantly attracting attention in exploring the unique properties of the N-doped 2D carbon family. In other words, the carbon-based 2D material will act as a support and also as an active electrocatalyst (Wan et al., 2015; Huang et al., 2016; Jiang et al., 2016; Wang et al., 2016; Zhang et al., 2016).

Recently, the synthesis method involving solution phase is emerged as one of the most potential methods leading toward precise control of the shape, size, and structure of metal nanoparticles, which could serve as promising ORR electrocatalysts (Khan et al., 2018b,c,d). Previously, in our lab, we productively schemed and generated a suitable one-step facile solution-based method to synthesize C12A7:e^−^ composite consisting of metal oxides and nitrogen-doped rGO materials (Khan et al., 2016, 2018a,b,c,d, 2019b; Zou et al., 2017). The synthesized composite has improved ORR performance to 0.1 M of KOH electrolytes for especially small-sized (~5-nm) nanoparticles with optimized crystalline structure and tuned electronic properties (Khan et al., 2018d).

Therefore, through this article, we slightly modified this synthesis scheme to prepare carbon-supported non-PGMs, Cr/rGO composite nanoparticles with/without MnO composite. In the next section, we will discuss the characterization-based result in detail.

## Synthesis and Characterization

### Materials Synthesis

**Figure 1:**
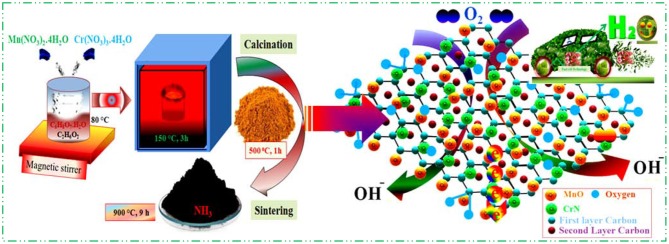
Scheme for Cr/rGO with MnO electrocatalyst synthesis and its proposed ORR application. rGO, reduced graphene oxide; ORR, oxygen reduction reaction.

Diverse synthetic approaches were schemed and applied to prepare the PGM-free electrocatalysts but had to face numerous challenges. Thus, we tried to overcome those limitations and synthesized nanosized metal oxides/nitrides composite with nitrogen-doped rGO by direct facile sol–gel method (Khan et al., 2018a,b,c,d, 2019b). Thus, a scalable preparation of crystalline from a molecular precursor followed by nitration under NH_3_ environment is reported here in Figure 1, where composite particles are shown in smaller size for more clarity.

### Synthesis of Cr/rGO Nanocomposite With MnO

First, the stoichiometric ratio of precursors Mn(NO_3_)_2_·4H_2_O and Cr(NO_3_)_3_·4H_2_O (1:1 and 0:1) was weighed. After that, raw materials were put in citric acid (CA) and ethylene glycol (EG) solution (4:1). To obtain xerox/dried gel, the mixture was heated slowly up to 150°C for 3 h. The resulting dried gel was crushed into powder and carefully heated at 400°C for 1 h in N_2_. It was again crushed into powder, and some part of the crushed powder was pressed to make the pellet at 150 MPa, and some powder part of as-synthesized product was sintered together at 900°C for 9 h in an alumina crucible under NH_3_ atmosphere, with a heating/cooling rate of 4°C·min^−1^, and Cr/rGO nano-composite with MnO was obtained. As a comparison, Cr/rGO composite without MnO composite was prepared by the same synthesis method only in the absence of Mn(NO_3_)_2_·4H_2_O.

### Materials Characterization

From precursors to final product, characterizations were done by applying diverse experimental techniques under different conditions. First, possible chemical reactions during sintering and thermal stability after sintering were determined using thermogravimetric/differential thermal analyses (TG-DTAs) (Diamond TG-DTA; Perkin-Elmer instrument (SII) Thermoplus, Rigaku). The temperature effect on required phase formation and its crystallinity were investigated by X-ray powder diffraction (XRD) using D8 Advanced Bruker AXS diffractometer with Cu-Kα radiation source (λ = 0.154 nm, 40 kV, 40 mA). Microstructural analyses of the final product were made using scanning electron microscopy (SEM) and transmission electron microscopy (TEM), along with element presence tested by energy-dispersive X-ray (EDX) mapping. The specific surface area (SSA), pore volume, and mean pore size measurements were carried out using the Brunauer-Emmett-Teller (BET) by Accelerated Surface Area and Porosimetry System (ASAP 2420). The atomic percent and weight percent of elements in each catalyst were obtained using an elemental analyzer (TruSpec Micro, Leco) and inductively coupled plasma optical emission spectrometry (ICP-OES-700-ES, Varian). An X-ray photoelectron spectrometer (XPS) ESCALAB 250Xi, which uses Al Kα (1,486.6 eV) X-rays as the excitation source, was used for elemental composition. C1s (284.8 eV) was selected as a reference.

Finally, regarding the application, electrocatalytic properties were studied. The electrocatalytic activities of the electrocatalysts were calculated by using cyclic voltammetry (CV) and rotating disk electrode (RDE) by CHI 760C analyzer. In the study of the ORR activity, first, the ink of synthesized composite was prepared. The powder was put in a mixture of H_2_O, isopropyl alcohol, and Nafion with volume ratios of 1:9:0.1 and sonicated for 30 min. Five microliters of prepared ink was uniformly casted onto a glassy carbon electrode (GCE) by simple dropping on the electrode with its apparent surface area of ~0.196 cm^−2^ and dried at room temperature. The smoothness of coated film on GCE surface is very important. The active mass loaded by the catalyst on the working electrode for all synthesized composites was ~0.66 mg·cm^−2^. In all measurements, the current normalization was performed using the GCE area, and its implication would be implied. The CV analysis was carried out at ambient conditions in O_2_-saturated (for 30 min) alkaline medium (0.1 M of KOH) and maintained over electrolyte surface during ORR testing to sustain the O_2_ saturation at potential cycling between 0.05 and 1.2 V vs. reversible hydrogen electrode (RHE) at 100 mV·s^−1^ until stable voltammogram curves were obtained. In alkaline ORR measurements, the prepared ink of 20% Pt/C was dropped on the glassy carbon RDE, yielding an approximate loading of 0.10 mg·cm^−2^, in which the contained loaded quantity of Pt is ~20 μg·cm^−2^. Next, for the RDE analysis, background capacitive currents were measured in a potential range of 1.2–0.2 V vs. RHE in N_2_-saturated electrolyte (scan rate of 10 mV·s^−1^). Finally, linear sweep voltammetry (LSV) in O_2_-saturated system was calculated with varying rotation speeds by fixing the rotation speed from 400 to 2,500 rpm in each measurement. Only 1,600-rpm rotation was carried out for comparison with benchmark of 20% Pt/C.

### Measured Data Conversion Equations

For data evaluation, all the potential data vs. saturated calomel electrode (SCE) obtained in this study were converted to potential vs. RHE by Equation (1):

(1) $$\begin{array}{r} E\left(\text{vs}.RHE\right)\;=\;E\left(\text{vs}.\;SCE\right)+\;E_{SCE}+0.0591\text{ pH}. \end{array}$$

The percentage of peroxide (% ${\text{HO}}_{2}^{-}$) during the ORR process was obtained by Equation (2):

(2) $$\begin{array}{r} \%{{\text{H}}_{2}\text{O}}^{-}\;=\;200\times \left(\frac{\frac{I_{r}}{N}}{{\frac{I_{r}}{N}+I}_{d}}\right) \end{array}$$

The electron transfer number and kinetics of ORR from rotating ring disk (RRD) measurement were evaluated by the Koutecky–Levich (K-L) equation,

(3) $$\begin{array}{r} \frac{1}{j}\;=\;\frac{1}{j_{l}}+\;\frac{1}{j_{k}}\;=\;\frac{1}{B\omega^{0.5}}+\frac{1}{j_{k}} \end{array}$$

(4) $$\begin{array}{r} B\;=\;0.64nFAC_{0}D_{0}^{2/3}V^{-1/6} \end{array}$$

(5) $$\begin{array}{r} J_{k}\;=\;nFkC_{0} \end{array}$$

where *J* is the measured current density; *J*_*l*_ and *J*_*k*_ are the limiting and kinetic current densities; *B* is the slope of K-L plots; ω is the rotation rate of the disk electrode; *n* is the electron transfer number in ORR; *F* is Faraday constant (96,485 C·mol^−1^); *A* is the geometric area of electrode (0.196 cm^2^); *C*_0_ is concentration of O_2_, which is 1.2 × 10^−6^ mol·cm^−3^ in 0.1 M of KOH; *D*_0_ is O_2_ diffusion coefficient (1.9 × 10^−5^ cm^2^·s^−1^); *V* is the kinematic viscosity of solution (0.01 cm^2^·s^−1^); and *k* is the electron-transfer-rate constant.

### Fuel-Cell Test

The synthesized Cr/rGO nanocomposite with MnO was used as a fuel-cell cathode to check its performance in the real system, judged through the membrane electrode assembly (MEA) analysis in the AAEMFCs. So, first, the synthesized catalyst and commercial ionomer (50 wt.%) were well dispersed in isopropyl alcohol via sonication to obtain homogeneous ink. Subsequently, the ink of a catalyst as a cathode and Pt/C as an anode was loaded on the active area of the gas diffusion layer. The MEA was fabricated by sandwiching potassium hydroxide-doped Tokuyama membrane between cathode and anode. Finally, the MEA was fitted in a single cell mode, which comprises serpentine flow field channels in graphite plates. The cell voltage and power were measured at 60°C, under 100% relative humidity and hydrogen and oxygen flow rates of 200 cc·min^−1^ (Sultan et al., 2018). The detailed analysis for material characterization to electrochemical analysis will be discussed in detail in the following sections.

## Results and Discussion

### Thermal Analysis

**Figure 2:**
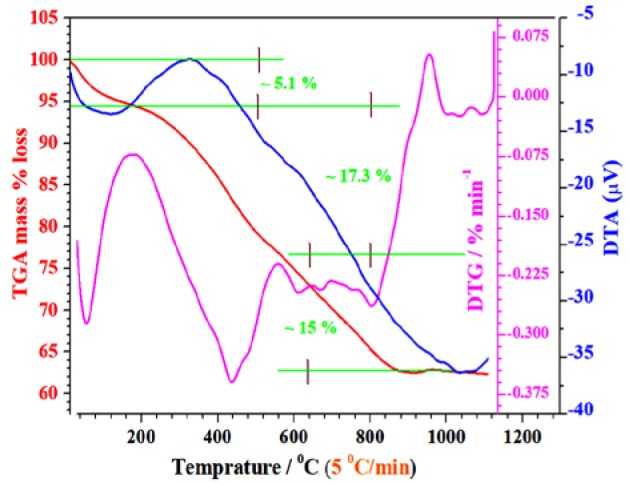
TG/DTA data of xerox gel form precursors. TG-DTA, thermogravimetric/differential thermal analyses.

TGA data of precursors of the Cr/rGO nanocomposite with MnO were collected by heat treatment of xerox gel precursor. The TGA causes phase transformation through the exothermic or endothermic process in the N_2_-gas environment from 30 to 1,100°C (Figure 2). The first weight loss was about 5%, which came into view possibly because of the physical absorption and because dehydrated crystalline water in the nitrates evaporated at about 100–150°C along with decomposition of precursor nitrides (Dong et al., 2014).

The second obvious continuous weight loss in the TG curve and corresponding exothermal peaks around 150–890°C is possibly because of the decomposition and burning of the loosely bonded organic species like nitrates and formation of required phase. After 890°C, no weight loss occurred, showing that the synthesis temperature higher than 890°C is suitable for synthesizing the required phase, so we selected 900°C.

### X-Ray Powder Diffraction Method

**Figure 3:**
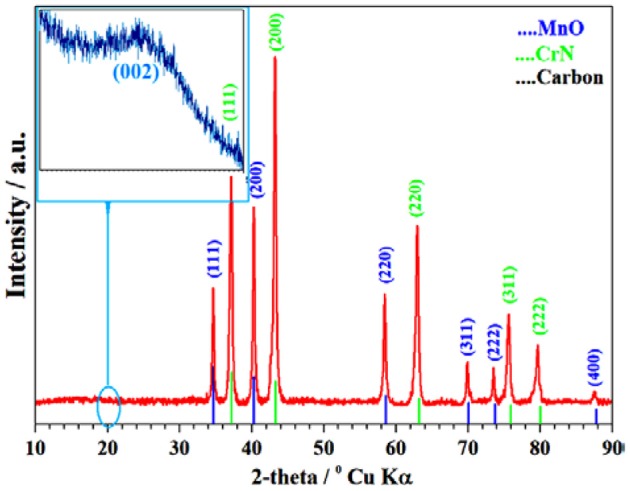
XRD of Cr/rGO nanocomposite with MnO. XRD, X-ray powder diffraction; rGO, reduced graphene oxide.

First, phase identification from XRD analysis was carried out (Figure 3). The Cr/rGO composite with MnO sample heat treated at 900°C, 9 h under NH_3_ environment, shows well-defined peaks of the MnO (JCPDS: 01-072-1533) and CrN (JCPDS: 03-065-2899) phases along with a small peak from carbon family. The carbon peak in the XRD pattern at about 23°Can be assigned as the typical graphitic (002) plane (Zhou et al., 2011). For further confirmation of this carbon peak, we first did Raman spectroscopy. Moreover, for further support, we performed a morphological study by high-resolution TEM (HR-TEM) and XPS of the Cr/rGO nanocomposite with MnO.

### Raman Analysis

**Figure 4:**
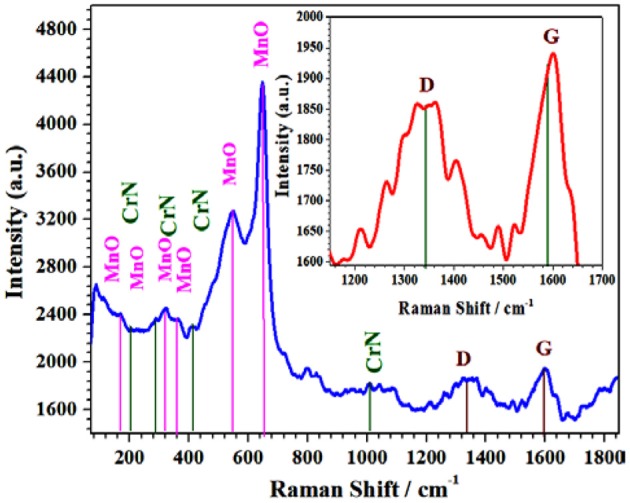
Raman spectra of Cr/rGO composite with MnO. rGO, reduced graphene oxide.

The Raman analysis of the Cr/rGO composite with MnO was carried out at 532-nm excitation wavelength using argon ion laser (20 mW) source (Figure 4). The 100–1,100 cm^−1^ Raman bands indicate the CrN and MnO lattice matching, and the 1,300–1,700 cm^−1^ is due to the carbon family, probably by rGO (Khan et al., 2018b,c).

For further proof about the reduction and formation of the rGO, Raman spectra of the samples from 1,100 to 1,800 cm^−1^ were recorded. In the inset in Figure 4, the two distinct peaks that appeared at 1,593 and 1,342 cm^−1^ correspond to the G-band peak and the D-band peak, respectively, for sp^2^-hybridized carbon. The D-band indicates the presence of disordered carbon, whereas the G-band indicates the presence of sp^2^-hybridized graphitic carbon. The D/G-bands have peak intensity ratio of about 0.95, which supported the formation of rGO (Mondalab et al., 2014). Hence, it confirmed the formation of Cr/rGO nanocomposite with MnO. For further verification, morphology of the sample was studied by applying SEM and TEM/HR-TEM analysis (Khan et al., 2018b,c,d).

### Scanning/Transmission Electron Microscopy

**Figure 5:**
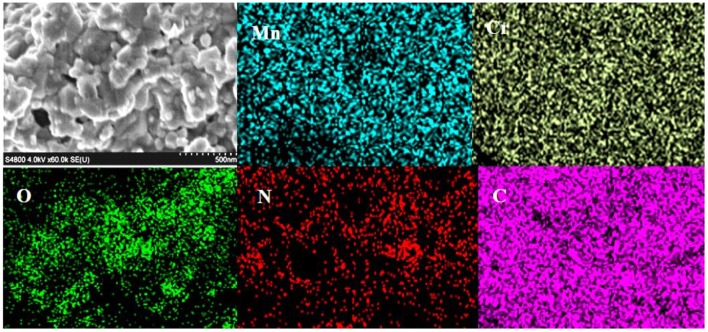
SEM image and EDX mapping of Cr/rGO nanocomposite with MnO, synthesized at 900°C in 9 h. SEM, scanning electron microscopy; EDX, energy-dispersive X-ray.

**Figure 6:**
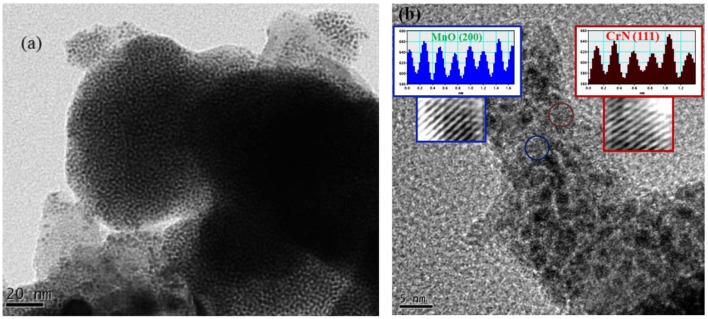
**(a,b)** TEM images of Cr/rGO nanocomposite with MnO, synthesized at 900°C in 9 h. TEM, transmission electron microscopy; rGO, reduced graphene oxide.

For surface morphological study of the Cr/rGO composite with MnO, before an electrocatalyst measurement, first, we studied the SEM (Figure 5). The SEM analysis shows nanosized particles, and the EDX mapping confirmed all expected elements in the Cr/rGO composite with MnO, that is, Mn, Cr, N, O, and C. For further clarification about the carbon present in the sample before an electrocatalyst measurement, exact particle size and surface morphology were studied by TEM/HR-TEM of synthesized sample. Figures 6a,b show that the particles have almost uniform size of about 4 nm (Sun et al., 2017). In Figure 6b, the HR-TEM also clearly shows that the MnO and CrN particles were present. The TEM results for the Cr/rGO composite without MnO are shown in the Supplementary Data (Figure S1), where the particle size is about 10 nm. No agglomeration occurs, which confirmed that the presence of the rGO is the basic reason in the case of Cr/rGO composite without/with MnO not to agglomerate them and hence obtained nanosized particles.

### Brunauer–Emmett–Teller Calculations

**Figure 7:**
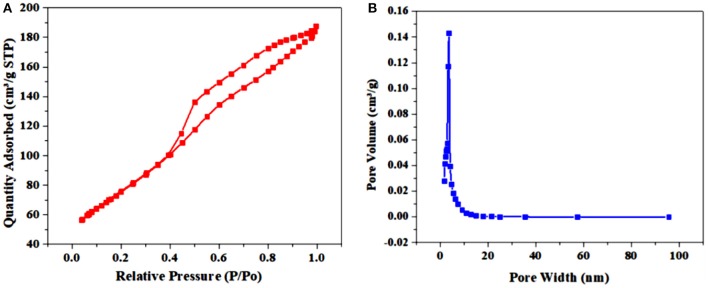
**(A)** N_2_ adsorption/desorption isotherms. **(B)** BJH curve of Cr/rGO nanocomposite with MnO. BJH, Barrett–Joyner–Halenda.

The surface analysis is critical for the ORR electrocatalysts measurements, so surface analysis was made using the BET analysis (Figure 7). The calculated BET surface area of the Cr/rGO composite with MnO was 379 m^2^·g^−1^, where its surface area is superior over carbon black support (216 m^2^·${\text{g}}_{\text{cat}}^{-1}$) (Figure 7A) (Bampos et al., 2017). Another important parameter is pore size estimation, which was estimated using Barrett–Joyner–Halenda (BJH) method from the adsorption branches for the synthesized Cr/rGO composite with MnO. Figure 7B discloses almost a single sharp peak at ~4 nm, proposing that the sample has almost homogeneous pore size distribution. It was found to be a type IV isotherm after examining the N_2_ adsorption desorption isotherm (Ranjbar and Rezaei, 2013). The highest surface area maybe because of the rGO, which not only enhanced the surface area but also did not allow the particles to agglomerate, also shown by TEM. Next, for further confirmation of the Cr/rGO composite with MnO, we performed an XPS analysis.

### XPS Study of Synthesized Powder

**Figure 8:**
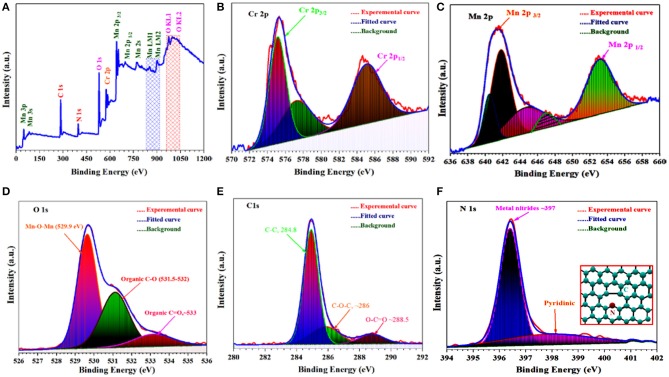
Full wide-scan XPS data of Cr/rGO nanocomposite with MnO. **(A)** High resolution. **(B)** Cr2p. **(C)** Mn2p. **(D)** O1s. **(E)** C1s. **(F)** N1s. XPS, X-ray photoelectron spectrometry; rGO, reduced graphene oxide.

Besides the Raman spectroscopy, the XPS was also performed to confirm the rGO formation and different elements within synthesized materials because the ORR activity is considered to stem from doping of the N into carbon structures in materials. Thus, to examine the chemical composition and oxidation states of different elements in as-synthesized Cr/rGO composite with MnO, the XPS analysis was carried out. The XPS was proved to be an effective way to determine chemical bonding nature of the N, C, Mn, O, and Cr species in the electrocatalysts. Figure 8A shows the XPS spectrum wide-range scan with all expected elements, showing the Mn2p, Cr2p, O1s, C1s, and N1s region spectra of Cr/rGO composite with MnO. The XPS wide-scan graph confirms that all the required/expected peaks were presented in the resulting graph. For further confirmations, we also studied all the elements by the XPS separately.

The Cr 2p XPS region (Figure 8B) was fitted with a component consisting of three peaks, at binding energies of 575.5, 577.5, and 585.5 eV, consistent with previously reported Cr phases (Watts et al., 2015). The binding energy separation of ~11.9 eV between the peaks at 653.2 and 641.2 eV is attributed to the Mn2p_1/2_ and Mn2p_3/2_, respectively (Figure 8C). The MnO has a satellite feature (~647 eV), which is not present for either Mn_2_O_3_ or MnO_2_ (Figure 8D) (Le et al., 2016). The pyridinic N species (N bonded to two carbon atoms) exhibit an N1s binding energy peak at ~398.1 eV (Wang et al., 2018), as shown in Figure 8F. Finally, for further analysis, the C1s peak was fully analyzed (Kondo et al., 2015). The C1s deconvoluted peak comprises in two peaks that are assigned to C–C and C = C bonds (~284.8 eV) and C–N bond (~286.01 eV). At 287.8 eV, a small peak was also observed, indicating O=C-N presence (Figure 8E) (Kumar et al., 2015). Elemental analyzer and ICP-OES (Table 1) confirmed all the required elements in Cr/rGO composite with MnO.

**Table 1:** The atomic percentage of each element obtained from ICP-OES of Cr/rGO composite with MnO.

**Catalysts**	**C (atomic %)**	**N (atomic %)**	**O (atomic %)**	**Mn (atomic %)**	**Cr (atomic %)**
Cr/rGO composite with MnO	07.93	8.01	19.01	33.03	32.02
Cr/rGO composite without MnO	12.62	14.03	17	00.00	56.35
Cr/Mn composite	00.00	10.20	11.30	41.10	37.40

*ICP-OES, inductively coupled plasma optical emission spectrometry; rGO, reduced graphene oxide*.

Hence, XPS (Table S1) with the ICP-OES result confirmed the Cr/rGO nanocomposite with MnO, which was also supported by the XRD and TEM results. The presence of the N and C determined with a Cr–Mn-based catalyst would be expected for the high density of CrN, MnO, and rGO active sites for ORR.

### Electrochemical Analysis

**Figure 9:**
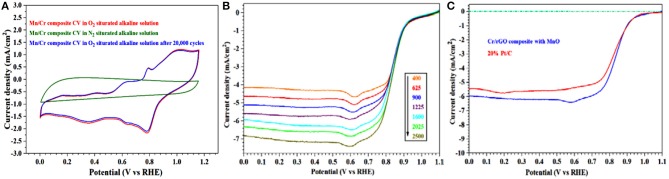
Cr/rGO nanocomposite with MnO. **(A)** CV curves obtained at a scan rate of 50 mV·s^−1^ in O_2_- and N_2_-saturated 0.1 M KOH solution. **(B)** LSV curves in O_2_-saturated 0.1 M KOH with various rotation rates. **(C)** Comparison of LSV curves of synthesized composite and those of commercial 20% Pt/C at rotation rate of 1,600 rpm. rGO, reduced graphene oxide; CV, cyclic voltammetry; LSV, linear sweep voltammetry.

Electrocatalysts facilitating the ORR are very important components in the advanced fuel cells and metal-air battery technologies. To evaluate the electrochemical performance of the Cr/rGO composite with/without MnO, toward the ORR activity, we checked the electrochemical activity by the CVs and RDE experimental calculations in a three-electrode (GC working electrode, graphite rod as counter electrode, and Hg/HgCl reference electrode) electrochemical cell performed, using different rotation rates (400–2,500 rpm). During the ORR measurement, no extra carbon black was added to improve the conduction of the samples. For the electrochemistry measurement, the electrode was first submerged in the 0.1 M KOH electrolyte and then tested over a particular suitable potential range. After 10,000 CV cycles, when the as-synthesized electrocatalyst CV curves become stable, we selected the final stable CV for further calculations. The CV test was used to determine the possibility of using the materials as catalysts for the ORR. The CV curves were obtained in the O_2_-saturated 0.1 M KOH (Figure 9A) of the Cr/rGO nanocomposite with MnO, showing a large cathodic reduction current peak at 0.8 V, which demonstrates its effective ORR activities in the alkaline media. On the other hand, the CV obtained in nitrogen-saturated electrolyte shows no reduction peak. Figure 9A also shows the typical CV curves of as-synthesized Cr/rGO composite with MnO, where the CV obtained after 20,000 cycles is also almost constant.

**Figure 10:**
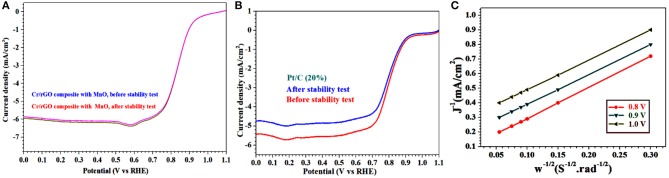
**(A,B)** LSV curves of Cr/rGO composite with MnO and 20% Pt/C before/after 20,000 potential cycles in O_2_-saturated 0.1 M KOH and at 1,600 rpm. **(C)** K-L plots of Cr/rGO composite with MnO at different potentials. LSV, linear sweep voltammetry; rGO, reduced graphene oxide; K-L, Koutecky–Levich.

The CV area of the Cr/rGO nanocomposite with MnO is also much greater, indicating that it has a large surface electrochemical active area, which is directly proportional to its SSA and shows good agreement with the BET results. To gain further insight into these acquired positive aspects, we collected the LSV curves of the Cr/rGO nanocomposites with MnO, which were investigated as electrocatalysts for the ORR in the 0.1 M KOH alkaline solution. The ORR polarization curves at the range of 400–2,025 rpm were obtained for investigating the ORR catalytic activity pathway of a synthesized material. The RDE polarization plots reflected catalytic activities of the Cr/rGO composite with MnO (Figure 9B). The ORR kinetics was evaluated from RDE analysis at particular rotation speeds of between 400 and 2,025 rpm. Kinetic values cleared the limited current density that augments as rotation rate increases. It was normally observed, because at higher speed, the diffusion distance of the O_2_-saturated solution is shortened, improving the mass transfer at the electrode surface (Khan et al., 2018d). It also demonstrates that the well-defined platform of diffusion-limited currents at all rotation speeds improves mass transport efficiently. The onset potential of the Cr/rGO composite with MnO is more positive than or as high as 1.05 V, nearly 50 mV higher than that of the 20% Pt/C (1.02 V) (Figure 9C) (Sultan et al., 2018). The RDE data show that the half-wave potential and current density of Cr/rGO nanocomposite with MnO were 0.89 V and 5.90 mA·cm^−2^, respectively, greater than those of the benchmark 20% Pt/C electrode (0.84 V and 5.50 mA·cm^−2^). Similarly, the RDE data show that the half-wave potential and current density of the Cr/rGO composite without MnO were 0.84 V and 5.50 mA·cm^−2^, respectively, which were similar to those of the state-of-the-art Pt/C electrode on carbon black (0.84 V and 5.50 mA·cm^−2^) (Figure S2) (Khan et al., 2016, 2018a,b,c,d, 2019b; Zou et al., 2017). Hence, the Cr/rGO composite without/with MnO, synthesized by this newly introduced method, has good ORR half-wave potential and current density, as we used the same synthesis method for synthesizing the C12A7:e^−^-rGO nanocomposite with good ORR results, which were previously reported (Khan et al., 2018d). The next important aspect regarding the ORR is the stability of the electrocatalyst. The sample was cycled from 0 to 1.2 V at 50 mV·s^−1^ in the O_2_-saturated 0.1 M KOH solution. The Cr/rGO composite with MnO exhibits excellent stability (Figure 10A). After 20,000 continuous cycles, the half-wave potential of the Cr/rGO nanocomposite with MnO is almost maintained. In contrast, a ~20-mV shift has been observed for the benchmark 20% Pt/C catalyst (Figure 10B).

The number of transfer electrons in the ORR was calculated from the K-L slope (Yuan et al., 2017; Khan et al., 2018d). The K-L slopes were plotted from the RDE analysis for the Cr/rGO nanocomposite with MnO (Figure 10C), which are almost linear near the parallelism, indicating that the kinetic is a first-order reaction toward the dissolved oxygen concentration in the solution. The number of electron transfer for the ORR was about 3.98–3.99 and for the potential 0.8–1.0 V, so it follows the four-electron system (Li et al., 2017; Wei et al., 2017; Yuan et al., 2017; Khan et al., 2018d). This four-electron nature shows that it can eliminate the losses because less or no corrosion takes place, which is the main reason for high durability of the synthesized Cr/rGO composite with MnO sample (Li et al., 2017; Wei et al., 2017; Yuan et al., 2017; Khan et al., 2018d).

**Figure 11:**
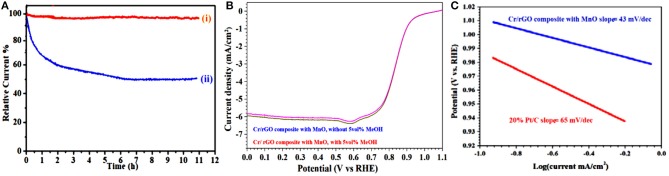
**(A)** Chronoamperometric response at 0.6 V of (i) Cr/rGO nanocomposite with MnO and (ii) Pt/C. **(B)** LSV curves of Cr/rGO nanocomposite with MnO, without/with 1 M of MeOH. **(C)** Tafel plots calculated from RDE polarization curves. rGO, reduced graphene oxide; LSV, linear sweep voltammetry; RDE, rotating disk electrode.

Robustness and impedance to fuel molecule (e.g., methanol) have prospective industrial significance in the alkaline fuel-cell industrialized technology. Therefore, we further compare the durability of the synthesized sample with the benchmark 20% Pt/C electrode, at the rotation rates of 1,600 rpm. The stability test (chronoamperometric) of the Cr/rGO nanocomposite with MnO and the Pt/C (20%) on GC electrode is carried out for 11 h in oxygen-saturated 0.1 M KOH electrolyte at 1,600-rpm rotation rate (Figure 11A) (Yuan et al., 2017; Khan et al., 2018d). After 11 h, the relative current of the Cr/rGO nanocomposite with MnO was almost constant (Ren et al., 2017), but a drop of about 60% was observed for the commercially bought benchmark 20% Pt/C electrode (Yuan et al., 2017). Furthermore, small variation in the LSV curve of the Cr/rGO nanocomposite with MnO was observed after methanol addition (Figure 11B) (Chen et al., 2015; Yuan et al., 2017). Hence, this behavior shows the potential application of the Cr/rGO nanocomposite with MnO as an electrode material in direct methanol fuel cells.

As we know, the Tafel slope is a kinetic parameter that gives information relating to the reaction mechanism. In addition, on high area catalysts evaluated by employing the thin-film RDE method, the Tafel slopes can also give information regarding the mass-transport conditions inside catalysts or even catalysts' conductivity. Therefore, we also measured the Tafel slopes. Figure 11C shows that the Cr/rGO nanocomposite with MnO exhibits ≈43 mV·dec^−1^ of Tafel slope, much smaller as compared with that of the commercially available benchmark 20% Pt/C (≈65 mV·dec^−1^), demonstrating that it is a highly active electrocatalyst. This high activity of the Cr/rGO nanocomposite with MnO is ascribed to the alloying transition of metals and nitrogen-doped rGO, which persuade favorable changes in the density of state (DOS) of metallic sites and in the activity (Khan et al., 2018d; Sultan et al., 2018).

The change in DOS and defects due to the extrinsic doping in the rGO cause enhancement in the catalytic activity. Moreover, another importance of the graphitic layer is that it efficiently confines synthesized material nanoparticles in a limited gap to keep away from agglomeration. The Cr/rGO composite without/with MnO exhibits higher activity accompanied by not only superior stability but also superior benchmark Pt/C with respect to the ORR current density, onset, and half-wave potential in the alkaline medium. We also compared the obtained electrochemical results in this study with some of previously published results, and our electrocatalytic results show priority over previous ones (Table 2).

**Table 2:** Electrocatalytic properties comparison.

**Catalyst**	**Onset potential (V)**	**Half-wave potential (V)**	**Current density (mA·cm^**−2**^)**	**References**
CrN/GC	0.86	0.66	3.50	Zhao et al. (2015)
α-MnO_2_	0.79	0.76	5.90	Chen et al. (2018)
N-rGO	0.84	0.83	2.25	Liang et al. (2012)
MnCo_2_O_4_/rGO	0.845	0.86	2.75	Liang et al. (2012)
Pt/C	0.95	0.82	5.40	Khan et al. (2018d)
Cr/rGO without MnO	1.03	0.84	5.50	This work
Cr/rGO with MnO	1.05	0.89	5.90	This work

*rGO, reduced graphene oxide*.

**Figure 12:**
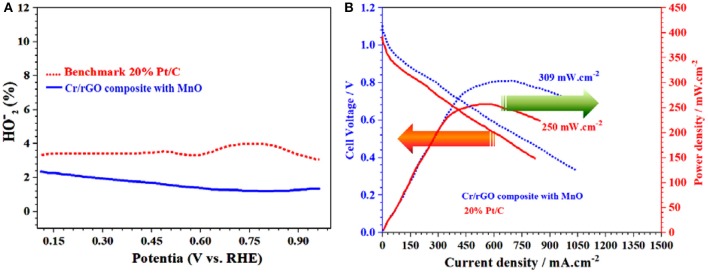
Cr/rGO nanocomposite with MnO. **(A)** ${\text{HO}}_{2}^{-}$ % yield for oxygen reduction reaction. **(B)** Polarization and power density curves of anion exchange membrane fuel cells. rGO, reduced graphene oxide.

After the electrocatalytic test, the TEM and the XRD of the Cr/rGO with MnO catalytic sample showed almost the same particles sizes (Figure S3) as well as phase (Figure S4). Hence, it is clear that there are no prominent changes observed in the crystalline phase and the morphology of the synthesized Cr/rGO composite with MnO, before and after electrocatalyst measurements. In short, this new catalyst has its novel electrocatalyst ORR properties that govern the importance for energy applications. The ${\text{HO}}_{2}^{-}$ % yield for ORR measured by the RRD electrode (RRDE) method of the Cr/rGO with MnO shows less yield as compared with the benchmark 20% Pt/C (Figure 12A).

Finally, regarding the industrial application, the polarization and power density curves of the H_2_/O_2_ AEMFC with the Cr/rGO nanocomposite with MnO cathode catalysts were obtained using A201-Tokuyama membrane, at cell temperature of 60°C, relative 100% humidity of the anode and cathode, H_2_ and O_2_ flow rate of 200 ml min^−1^, and back-pressure of anode and cathode of 30 psi (Figure 12B) (Sultan et al., 2018). The open circuit voltage of Cr/rGO with MnO-based AEMFCs was 1.05 V, which is almost equal to LSV-based calculated onset potential. Also, the acquired maximum power was 250 mW·cm^−2^ and 309 mA·cm^−2^ for the benchmark 20% Pt/C and the Cr/rGO nanocomposite with MnO, respectively. Hence, the performance of the Cr/rGO nanocomposite with MnO-based AEMFCs was higher than that of the benchmark 20% Pt/C-based AEMFCs (Xin et al., 2013; Sultan et al., 2018).

## Conclusion

In this study, we designed a facile synthesis strategy regarding synthesizing highly active non-PGM-based catalysts along with further modulation in size, which boosts the electrocatalytic characteristics of this non-Pt-based catalyst. The Cr/rGO nanocomposite with MnO catalyst has half-wave potential and limited current density of 0.89 V and 5.90 mA·cm^−2^, respectively, higher than those of the benchmark 20% Pt/C (0.84 V, 5.50 mA·cm^−2^). Many factors can cause this increase in current density. Firstly, this increase in current density may be because of the sufficient metallic nature of synthesized, Cr/rGO composite with MnO, which favors faster electron movement and better adsorption of oxygen molecules on the catalyst surface. The Cr/rGO nanocomposite with MnO acquired maximum power density of 309 mA·cm^−2^ and proved to be a potential electrocatalyst candidate for the ORR, with high robustness. It has the advantage of cost-effectiveness and large-scale availability. The design scheme developed for the synthesis of the ORR active material can also be extended to other transition-metal-based composites as well as other applications in the electrochemical sensing and energy storage.

## Data Availability Statement

All datasets generated for this study are included in the article/Supplementary Material.

## Author Contributions

KK gave the idea. AT and MA wrote the paper. QaK, RW, SK, AS, ZG, QuK, YZ, ZO, and HZ finalized the paper.

### Conflict of Interest

The authors declare that the research was conducted in the absence of any commercial or financial relationships that could be construed as a potential conflict of interest.
