# Supplementary material for: Novel Two-Dimensional Carbon–Chromium Nitride-Based Composite as an Electrocatalyst for Oxygen Reduction Reaction
Source: Front Chem. 2019 Nov 12;7:738. doi: 10.3389/fchem.2019.00738 (PMC6861161; doi:10.3389/fchem.2019.00738)
Supplement: Supplementary file 1 [file Data_Sheet_1.PDF]

Novel two dimensional carbon-chromium nitride based composite as an electrocatalyst for Oxygen Reduction Reaction

Karim Khan[1,3,6,*,+], Ayesha Khan Tareen[3, *,+], Muhammad Aslam[2,+], Qasim khan[4], Sayed Ali Khan[4], Qudrat Ullah Khan[4], Awais Siddique Saleemi[5], Renheng Wang[3], Yupeng Zhang[3], Zhongyi Guo[6], Han Zhang[3]*, and Zhengbiao Ouyang[1]*.

1. *College of Physics and Optoelectronic Engineering, THz Technical Research Center, Key Laboratory of Optoelectronics Devices and Systems of Ministry of Education and Guangdong Province, Shenzhen University, Shenzhen, 518060, P.R. China.*
2. *Government Degree college PaharPur, Gomel University, Dera Ismail Khan, K.P.K., Islamic Republic of Pakistan.*
3. *Shenzhen Engineering Laboratory of Phosphorene and Optoelectronics, and SZU-NUS Collaborative Innovation Center for Optoelectronic Science and Technology, Shenzhen University, Shenzhen, 518060, P. R. China.*
4. *Shenzhen Key Laboratory of Flexible Memory Materials and Device, Collage of Electronic Science and Technology, Shenzhen University, Nanhai Ave. 3688, Shenzhen, Guangdong 518060, P.R. China.*
5. *Key Laboratory of Optoelectronic Devices and Systems of Ministry of Education and Guangdong Provence, College of Optoelectronic Engineering, Shenzhen University, Shenzhen, Guangdong, 518060, P. R. China.*
6. *Advanced electromagnetic function laboratory, Donggun university of Technology, Donguan, Guangdong, P. R. China.*

Corresponding author: karim_khan_niazi@yahoo.com (***Karim Khan***), chemistayesha@yahoo.com (***Ayesha Khan Tareen***), hzhang@szu.edu.cn (**Han Zhang**), and zbouyang@szu.edu.cn (***Zhengbiao Ouyang***).

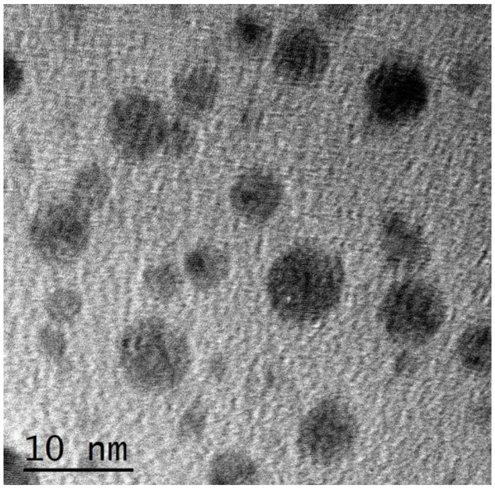

Fig.S1. TEM images of Cr/rGO composite without MnO synthesized at 900 ^{0}C, 9 h.

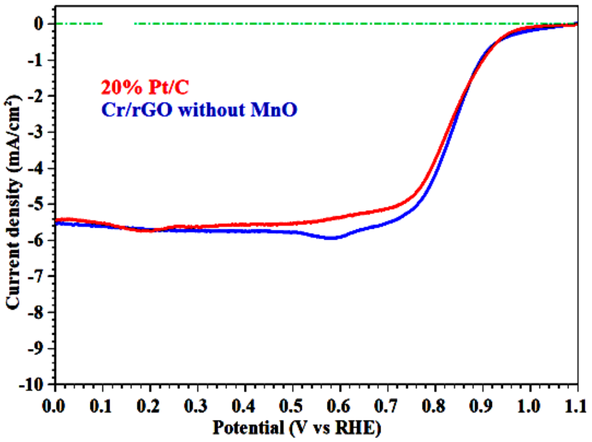

Fig.S2. Comparison of the LSV curves in O_2-saturated 0.1M KOH of, Cr/rGO without MnO and benchmark 20% Pt/C at 1600 rmp.

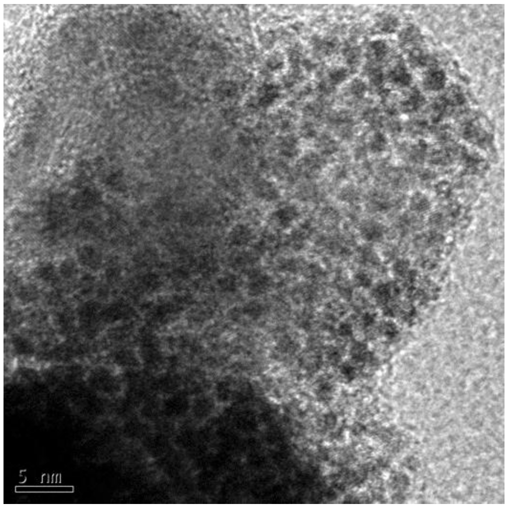

Fig.S3. TEM images of Cr/rGO nanocomposite with MnO, synthesized at 900 ^{0}C, 9 h after electrocatalytic analysis.

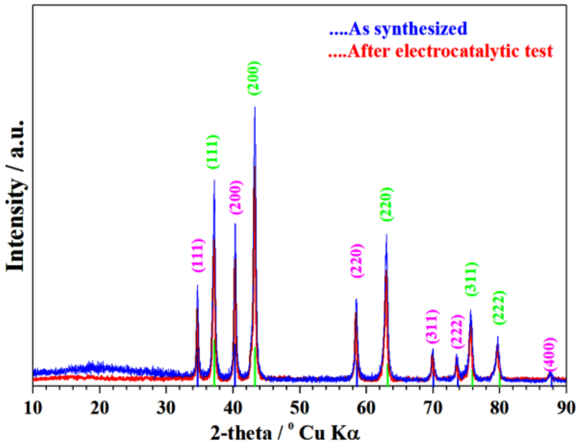

Fig.S4. XRD of nitrated Cr/rGO nanocomposite with MnO, synthesized at 900 ^{0}C, 9 h under NH_3 environment, before (blue) and after (red) electrocatalytic test.

Table S1. The XPS based elemental composition (atomic %) data obtained from Cr/rGO nanocomposite with MnO.

**Catalysts**	**C (Atomic %)**	**N (Atomic %)**	**O (Atomic %)**	**Mn (Atomic %)**	**Cr (Atomic %)**
Cr/rGO nanocomposite with MnO	08.83	7.98	18.01	34.01	31.17
